# Spatial and temporal avoidance of risk within a large carnivore guild

**DOI:** 10.1002/ece3.2616

**Published:** 2016-12-16

**Authors:** Egil Dröge, Scott Creel, Matthew S. Becker, Jassiel M'soka

**Affiliations:** ^1^ Department of EcologyMontana State UniversityBozemanMTUSA; ^2^ Zambian Carnivore ProgrammeMfuweEastern ProvinceZambia; ^3^ Department of National Parks & WildlifeChilanga, LusakaZambia

**Keywords:** carnivore, conservation, interspecific competition, intraguild predation, niche partitioning, risk effect

## Abstract

Within a large carnivore guild, subordinate competitors (African wild dog, *Lycaon pictus*, and cheetah, *Acinonyx jubatus*) might reduce the limiting effects of dominant competitors (lion, *Panthera leo*, and spotted hyena, *Crocuta crocuta*) by avoiding them in space, in time, or through patterns of prey selection. Understanding how these competitors cope with one other can inform strategies for their conservation. We tested how mechanisms of niche partitioning promote coexistence by quantifying patterns of prey selection and the use of space and time by all members of the large carnivore guild within Liuwa Plain National Park in western Zambia. Lions and hyenas specialized on wildebeest, whereas wild dogs and cheetahs selected broader diets including smaller and less abundant prey. Spatially, cheetahs showed no detectable avoidance of areas heavily used by dominant competitors, but wild dogs avoided areas heavily used by lions. Temporally, the proportion of kills by lions and hyenas did not detectably differ across four time periods (day, crepuscular, early night, and late night), but wild dogs and especially cheetahs concentrated on time windows that avoided nighttime hunting by lions and hyenas. Our results provide new insight into the conditions under which partitioning may not allow for coexistence for one subordinate species, the African wild dog, while it does for cheetah. Because of differences in responses to dominant competitors, African wild dogs may be more prone to competitive exclusion (local extirpation), particularly in open, uniform ecosystems with simple (often wildebeest dominated) prey communities, where spatial avoidance is difficult.

## 1 Introduction

Interference competition affects virtually all species (Pianka, 1981; Schoener, 1974; Sinclair, 1985; Ziv, Abramsky, Kotler, & Subach, 1993), and is widely recognized as an important force structuring large carnivore guilds (Caro & Stoner, 2003; Creel, Spong, & Creel, 2001; Palomares & Caro, 1999). The limiting effect of competition between large carnivores can be strong (Creel & Creel, 1996; Estes & Goddard, 1967; Mills & Biggs, 1993; Mills & Gorman, 1997), and to reduce these effects, subordinate species typically respond to dominant competitors through some combination of diet partitioning, spatial segregation or temporal segregation (Cozzi, Broekhuis, Mcnutt, & Schmid, 2013; Crooks, Van Vuren, & Crooks, 1995; Hayward & Slotow, 2009; Périquet, Fritz, & Revilla, 2014). In recent years, the role of large carnivores in shaping ecosystems and maintaining biodiversity has become increasingly clear (Baum & Worm, 2009; Ripple et al., 2014; Ritchie & Johnson, 2009), along with global declines in large carnivore populations (Estes et al., 2011). The ecological effects of terrestrial large carnivores remain strong in Africa relative to most other parts of the world, and large protected areas in Eastern and Southern Africa have retained ecologically intact large carnivore guilds including lion (*Panthera leo*), leopard (*Panthera pardus*), spotted hyena (*Crocuta crocuta*), cheetah (*Acinonyx jubatus*), and African wild dog (*Lycaon pictus*) populations.

All of these species prey primarily on ungulates, often with substantial dietary overlap, thereby creating the potential for exploitative or interference competition within the large carnivore guild (Creel & Creel, 1996; Hayward, O'Brien, Hofmeyr, & Kerley, 2006). Interference competition can be particularly strong because kleptoparasitism among these species is common, probably because it reduces the energetic costs of hunting by eliminating the costs of hunting and killing prey (although stealing carcasses also entails some costs and risks). Furthermore, the morphological adaptations used to kill large prey also present dangers in competitive encounters and increase the cost of direct interactions (Creel et al., 2001; Palomares & Caro, 1999). During direct interactions between species, lions and hyenas are dominant competitors, while cheetahs, leopards, and African wild dogs are subordinates. The two dominant competitors have both beneficial and detrimental effects on each other (Périquet et al., 2014). Each can steal kills from the other, each can kill the other, and the net effect on each other's population dynamics or fitness is often not obvious. Due to asymmetry in body size, cheetahs and wild dogs rarely kill lions or hyenas but are known to be killed by both (Caro & Laurenson, 1994; Creel & Creel, 1998), and lose kills to kleptoparasitism more often than they obtain them (Creel & Creel, 1996, 1998; Ginsberg et al., 1995). Consequently, the net effect of lions and hyenas on the dynamics of subordinate competitors is usually negative, as revealed by a negative correlation in the densities of subordinate and dominant competitors across ecosystems (Creel & Creel, 2002; Swanson et al., 2014) and by negatively correlated changes in density through time within ecosystems (Creel & Creel, 2002; Swanson et al., 2014).

Natural selection favors adaptations (including behavioral responses) that reduce the cost of competitive interactions. Mechanisms of niche partitioning have seen substantial prior study in African large carnivores (Broekhuis, Cozzi, Valeix, Mcnutt, & Macdonald, 2013; Cozzi, Broekhuis, Mcnutt, Turnbull, & David, 2012; Cozzi et al., 2013; Creel & Creel, 1996; Durant, 2000a; Hayward & Slotow, 2009; Vanak et al., 2013). As with antipredator responses of prey, carnivores can respond to the risk of costly competitive interactions proactively or reactively. Reactive responses typically take place on small spatial and temporal scales as a result of direct interaction between the species, and are likely to be clear‐cut (e.g., wild dogs fleeing after a direct encounter with lions). Proactive avoidance between animals utilizing the same resources takes place on larger spatial (significant part of home range size or larger) and temporal (seasonal or annual) scales, in response to cues of risk that are subtle and usually less obvious to the human observer (e.g., cheetahs avoiding locations commonly used by lions). When dominant competitors occupy areas of high prey concentration, proactive avoidance of dominant competitors can force subordinate competitors to trade food for safety.

Several studies have found that African wild dogs are negatively affected by hyena kleptoparasitism (Creel & Creel, 1996; Estes & Goddard, 1967; Gorman, Mills, Raath, & Speakman, 1998; Kruuk, 1972; Speakman, Gorman, Mills, & Raath, 2015), and studies in an overlapping set of ecosystems show that lions can limit African wild dogs in numbers and distribution, through a combination of direct predation and competitive exclusion from areas of high prey density (Creel & Creel, 1996; Ginsberg et al., 1995; Mbizah, Marino, & Groom, 2012; Vanak et al., 2013). Mills and Gorman (1997) found that African wild dogs in Kruger National Park avoided lions and thus did not favor habitats with high impala density, even though impala comprised 71% of their prey. Similarly, wild dogs in the Selous Game Reserve avoided areas heavily used by lions, and consequently hunted disproportionately often in habitats where they had significantly reduced rates of encounter with prey (Creel et al., 2001).

In contrast to these results for wild dogs, Vanak et al. (2013) found substantial spatial overlap in the ranges of lions and cheetahs (while confirming low overlap between lions and wild dogs). Broekhuis et al. (2013) examined the effects of both lions and hyenas on cheetahs. Similar to the results of Vanak et al. (2013), space use by cheetahs was highly similar to that of lions and hyenas over long timescales, but within these areas of shared use, cheetahs avoided risk on short timescales by positioning themselves further from the nearest lions and hyenas than expected by chance (Broekhuis et al., 2013). Durant (1998, 2000a, 2000b) also found that cheetahs avoided both lions and hyenas and concluded that ability to hunt within local “competition refuges” was critical for their persistence within an ecosystem.

In addition to (or alternative to) spatial avoidance, subordinate competitors can avoid dominant competitors temporally, by hunting when dominant competitors are less active. Darnell, Graf, Somers, Slotow, & Szykman Gunther (2014) found that in Hluhluwe–iMfolozi Park, African wild dogs showed both spatial and temporal avoidance of lions, particularly during denning periods, but did not show any avoidance of hyenas. Durant (1998) found that cheetahs avoided lions and hyenas in both space and time. In contrast, Cozzi et al. (2012) found a high degree of overlap in activity signals from GPS collars on African wild dogs, cheetahs, lions, and hyenas, concluding that overlaps in activity patterns were driven by food limitation that constrained avoidance in that ecosystem. Despite this result, data from most ecosystems show that the majority of hunts by wild dogs and (especially) cheetahs are typically diurnal or crepuscular (Creel & Creel, 2002; Estes & Goddard, 1967; Mills & Biggs, 1993), which reduces the likelihood of direct interference competition with more nocturnal lions and (especially) hyenas (Cozzi et al., 2012; Kolowski, Katan, Theis, & Holekamp, 2007; Mills & Biggs, 1993). While some authors suggest that crepuscular activity of primarily nocturnal carnivores could perhaps increase their hunting success (Hayward & Slotow, 2009), temporal partitioning of activity has generally been interpreted as a mechanism by which subordinate carnivores can reduce the frequency of both food loss and the risk of injury or death by direct interactions with lions and hyenas at contested kill sites.

As mentioned above, the relationship between the two dominant competitors, lions and hyenas, is a complex mixture of facilitation and competition (Périquet et al., 2014). In a comprehensive review of hyena–lion interactions, Périquet et al. (2014) found strong evidence for costs to both species through exploitation and interference competition through diet overlap, intraguild predation, and kleptoparasitism, but also identified benefits to both species from scavenging opportunities, and mechanisms that reduce costs such as differences in prey selection (Pereira, Owen‐Smith, & Moleón, 2014; Périquet et al., 2014). In contrast, Trinkel and Kastberger (2005) found no benefits of lions for hyenas.

Prior studies of competition within the large carnivore guild have often focused on a single pair of species (but see Broekhuis et al., 2013; Cozzi et al., 2012; Creel & Creel, 2002; Mills & Biggs, 1993; Vanak et al., 2013). Camera trapping studies have been used to study niche partitioning within broader guilds, mainly to analyze broad patterns of spatial overlap (Ngoprasert et al., 2012; Steinmetz, Seuaturien, & Chutipong, 2013). However, the frequency of detection on camera traps for most large carnivores is generally too low to meaningfully analyze patterns of spatial or temporal avoidance (Ngoprasert et al., 2012). For example, in an intensive camera trapping study to estimate leopard population density and survival, individuals known to be present often went undetected for a month or more (Rosenblatt et al., 2016), implying low power to detect patterns of interaction or spatial avoidance on the relevant timescale, and somewhat limited power to detect temporal avoidance. Spatial data from GPS collars can provide strong inferences about proactive spatial avoidance, but typically do not provide samples adequate for inferences about reactive avoidance (Creel, Winnie, & Christianson, 2013). Neither camera trapping nor GPS collars provide information on dietary overlap or partitioning. To simultaneously examine these three aspects of niche partitioning, one needs to couple location data with direct observation, which has rarely been accomplished for a complete large carnivore guild. The dataset in this study is unique because it couples extensive location data with extensive direct observations, and thus has good power to analyze long‐term spatial relationships, short‐term activity patterns, and dietary overlap/partitioning of all large carnivore species present within an ecosystem. Finally, inferences about spatial avoidance are often complicated by the difficulty inherent in determining whether differences in space use are due to competition or simply reflect differences in habitat selection that would occur without competitive constraints (Cozzi et al., 2013; but see Creel & Creel, 2002; Mills & Gorman, 1997): Because the ecosystem we studied is highly uniform with respect to habitat (Figure 2) and supports a simple ungulate guild dominated by only three species (M'Soka, 2015), the interpretation of spatial and temporal overlaps is simplified considerably.

We examined the use of space and time by the large carnivore guild of Liuwa Plain National Park (LPNP) in western Zambia, consisting of hyenas, lions, cheetahs, and African wild dogs. Leopards are absent from LPNP. Historically, they occupied woodland areas along rivers, but have not been recorded in LPNP for the last 50 years. Based on prior studies, we hypothesized that African wild dogs and cheetahs would avoid lions and hyenas in both space and time to reduce kleptoparasitism and direct predation. We hypothesized a negative relationship between space use of subordinate competitors (African wild dogs and cheetahs) and dominant competitors (lions and spotted hyenas). We hypothesized that subordinate competitors would make a majority of their kills during time periods in which dominant competitors were least active. We hypothesized that hyenas and lions would not show such patterns with regard to each other. Finally, we hypothesized that hyenas and lions would show strong dietary overlap by specializing on the most abundant prey and that cheetahs and wild dogs would reduce dietary overlap by preying on a wider range of species.

## 2 Methods

### 2.1 Study area and populations

**Figure 1:**
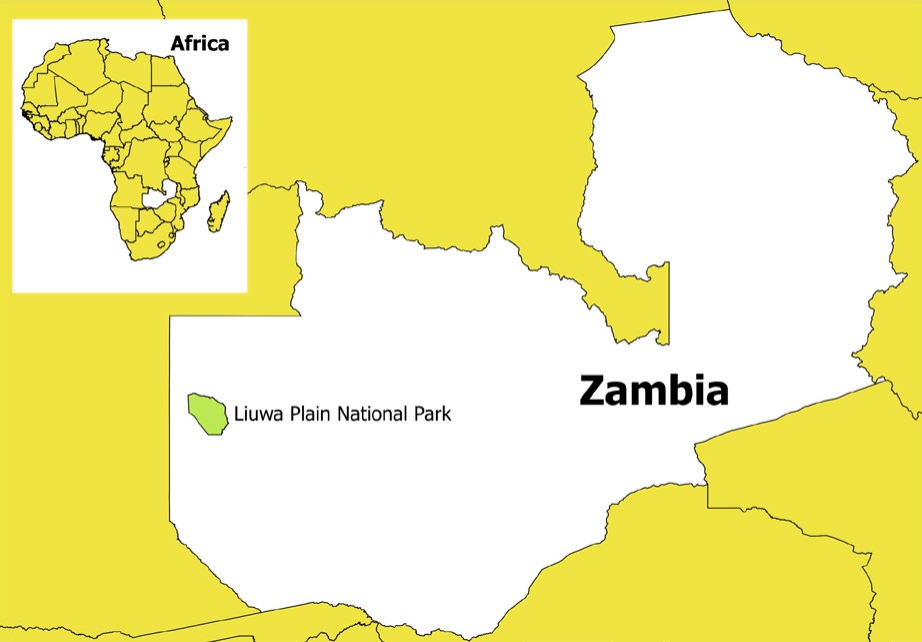
Location of Liuwa Plain National Park within Zambia and Africa

**Figure 2:**
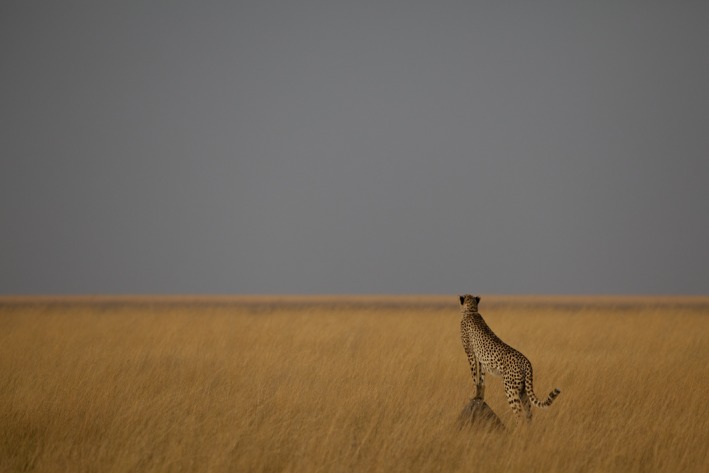
The 1,200‐km^2^ focal study area is dominated by open grasslands heavily used by migratory wildebeest and zebra and resident oribi. The homogeneity of habitat type and structure within our study area simplified interpretation of differences between species in their use of space

All of our data were gathered from a 1,200‐km^2^ study area within Zambia's 3,660‐km^2^ LPNP (Figure 1). This area holds Africa's second largest wildebeest migration, within LPNP and areas immediately northwest. Our study area is in the southern portion of LPNP, dominated by short and intermediate grasslands with occasional tree islands (Figure 2), and supports an ungulate community dominated by migratory wildebeest (*Connochaetes taurinus*) at densities ranging from 6.2 to 60.8 individuals/km^2^), migratory zebra (*Equus quagga*) at densities ranging from 1.8 to 8.1 individuals/km^2^, and nonmigratory oribi (*Ourebia ourebi*) at densities ranging from 1.1 to 14.5 individuals/km^2^. These densities were estimated from distance sampling on a systematic transect grid two to three times in each year of the study, with a total transect length of 1,280 km (M'Soka et al., 2016). Other species that were occasionally killed occurred at much lower densities, including common duiker (*Sylvicapra grimmia*), red lechwe (*Kobus leche*), steenbok (*Raphicerus campestris*), common reedbuck (*Redunca arundinum*), and scrub hare (*Lepus saxatilis*). Hyenas greatly outnumbered other carnivores within the study area, with populations of 151 hyenas in four clans (M'Soka, Creel, Becker, & Droge, 2016), six lions in one pride, 22 wild dogs in two packs, and 17 known cheetahs.

### 2.2 Location data

Locations of African wild dogs, hyenas, and lions were collected between 24 June 2010 and 23 December 2015 using GPS and VHF radio collars fit to individuals in all but one group known to reside wholly or largely within the study site (details below). From 07 January 2012 to 13 April 2013, the alpha female of a resident African wild dog pack was fitted with a GPS collar taking a location every 5 hr. A second resident pack was fitted with VHF collars and was monitored from 25 June 2010 to 30 May 2014 after which the last members of the pack left the study area permanently following the death of the alpa male and subsequent dissolution of the breeding pack. From 24 October 2010 to 13 May 2012, five hyenas (in three of the four resident clans) were fitted with GPS collars (recording a location every 2 or 3 hr). During the course of the study, one of these clans split into two, but frequent resightings of known individuals confirmed that the two newly formed clans remained largely within the source clan's area. The remaining clan was monitored with VHF collars on five clan members, which provided a total of 318 locations (two hyenas in this clan were fitted with GPS collars, but the GPS function failed in both). The lion population in LPNP was actively restored after only one lioness remained in 2009. Several females and males were reintroduced and a maximum of six lions were present in the study area during the study period, consisting of one coalition of two males, and one pride of females and cubs. Between 24 June 2010 and 28 May 2015, at least one male and one female were fitted with a GPS satellite collar or GPS remote download collar (recording a location every 3 or 4 hr). The lions formed cohesive groups (males were never recorded without each other), and the home ranges of males and females largely overlapped (see section 3). All cheetah locations came from observations from four cheetahs fitted with VHF collars between 02 August 2012 and 23 December 2015. These included single locations from opportunistic observations, and multiple locations from hunt follows in which cheetahs were observed continuously through complete periods of activity. When moving, a GPS location was recorded at 15‐minute intervals, and while stationary only one location was recorded, with the start time and end time at that location. Cheetahs were followed in 50 periods between 12 November 2012 and 23 December 2015 for up to 7 days. Although the methods for cheetahs provided fewer locations than GPS collars on the other species, this sampling was representative for cheetahs within the study site, as all known groups included a collar. Locations for all four species were used to fit utilization distributions (UDs) that were resampled in an identical manner for each species to compare space use (as described below). All immobilization procedures to fit animals with collars were conducted with permission, following animal welfare standards and protocols required by the Zambia Department of Veterinary and Livestock Services and the Department of National Parks and Wildlife. The location data for the four species come from overlapping periods from 2010 to 2015, but the interval sampled was not identical for all species. However, data from GPS collars show that the ranging patterns of lions and hyenas changed very little year to year. Thus, the species for which we have the most locations are the dominant competitors, both with temporally stable spatial distributions, providing a good basis for tests of avoidance by the subordinate competitors during the period that they were sampled.

### 2.3 Utilization distributions

Location data were used to calculated UDs for each species using the adehabitatHR package (Calenge, 2006) in R (R Core Team, 2016, version 3.1). The UD reflects the relative intensity of use for a location, represented by a grid cell, within the home range of a species or group of animals. We used a grid cell size of 1,000 m. This spatial scale was fine enough to resolve variation in space use, and we had enough locations for every species to examine utilization at this scale. Standard methods for bandwidth selection (e.g., least‐squares cross‐validation) did not converge, probably due to very large home range sizes for some, and the standard reference bandwidth (href) yielded rather discontinuous UDs. In such cases, several authors (Silverman, 1986; Wand & Jones, 1994) recommend using a subjective visually chosen bandwidth as smoothing parameter. We used a more objective approach by calculating the daily distance moved for each individual animal by summing the distances between consecutive locations within a day, for individuals fitted with GPS collars or observed during hunt follows. We then examined the frequency distribution of daily distances for each species and selected the 90th percentile (95th for cheetahs, with sparser data) as the bandwidth. This procedure yielded largely continuous UDs, occasionally keeping some clustered locations separate in a plausible manner. Because the home ranges of the two wild dog packs and the four hyena clans had little overlap, UDs were calculated separately for each group, and then combined and rescaled to result in a total utilization of 1 for the combined UD. This process properly resolved areas of low use between home ranges. For cheetahs and lions, individual ranges overlapped very substantially, so we calculated a single UD for each species.

To test for correlations in the use of space for each pair of species, we sampled the calculated UD for each species using a grid of points separated by 1,000 m in both dimensions. The starting point of the grid was generated randomly in a 25‐km^2^ area outside of the study area (oriented N–S). We selected 1,000‐m grid spacing to balance an unavoidable trade‐off between spatial autocorrelation and sample size. To ensure coverage of the study area, we preferred sampling the UDs with a grid, rather than random location, and tested whether our inferences were affected by the choice of grid cell size (they were not).

We recorded locations outside of our 1,200‐km^2^ study site for all species, but our analysis is based only on locations within the focal study area. This restricted the analysis to an area within which we had good data on utilization by all four species, avoiding problems of interpretation that arise with a patchy distribution of sampling effort (i.e., where low utilization can be an artifact of sampling). The UD values within the study area for each species were scaled from 0 to 1 for ease of interpretation as “relative use”. However, it should be recognized that absolute intensity of utilization, and thus the potential strength of interference competition, also depends on the population density of each species, and as noted above, hyenas greatly outnumbered the other carnivores.

In addition to restricting our analysis to the well‐sampled focal study area, we restricted our analysis to areas known to be used within the focal area. Thus, we extracted UD values for each carnivore species at each grid location and tested the correlations between species with data restricted to locations with nonzero values for both species. In this way, we tested for spatial avoidance in areas of shared use for all possible pairs except for wild dog and cheetah, which have not been observed to interact strongly in any ecosystem.

Following Zuur, Ieno, and Elphick (2009), we used quasibinomial generalized linear models fit with the glm function in R to test the relationship for each species pair, in a manner that accounted for extrabinomial variation. We assessed the fit of all models using Q–Q and scale–location plots, which confirmed good fits. Heteroscedasticity was apparent in most cases, but the effect of heteroscedasticity is to reduce power. Because we did detect effects where they were expected (see section 2.5), heteroscedasticity was “not a reason to throw out an otherwise good model” (Mankiw, 1990). Maps of the UDs were created in QGIS 2.10.1‐Pisa (QGIS Development Team, 2015). Our a priori hypotheses and thus our initial tests were for linear relationships, but we tested whether other plausible functional forms provided a better fit. For the relationships of African wild dogs, hyenas, and cheetahs to lions, drop‐in‐deviance tests supported second‐order polynomial and exponential relationships (see section 2.5).

### 2.4 Activity data

To analyze whether carnivores avoided each other temporally, we recorded the time at which kills were made by each species. For this analysis, we considered only kills that were directly observed and probable kills (judged to be less than one hour old, with only a single carnivore present and no evidence of attendance by other carnivores from spoor). We never detected lions losing kills. If a kill made within the hour by cheetahs or African wild dogs was lost to lions or hyenas, we were likely to detect them nearby, because all African wild dog packs and cheetah groups were collared. We never detected hyenas losing kills to cheetahs or African wild dogs. They did lose kills to lions, as detected by direct observation, presence at kills, and spoor. To obtain representative data for all species, hunt follows were conducted by direct observation over complete hunting periods. A hunt follow consisted of a complete follow of an animal from the time it started hunting to the time it stopped hunting. Often these follows were performed for several consecutive hunt periods for the same species. While not all species were followed for an equal number of hours, this affects only the precision of estimates of the proportion of kills made per time period (but does not produce bias): The precision obtained was adequate for all species (see section 2.5). There were a total of 453 carcasses with a well‐described time of death, between 03 December 2010 and 07 December 2015. Within our sample, 77.5% of kills were directly observed, with the remaining 22.5% judged to be less than one hour old, and carcasses judged to be older than one hour not included. Times of kills were grouped into four periods of the day, the crepuscular period (5–7 a.m. and 5–7 p.m.), daytime (8 a.m.–4 p.m.), early night (8 p.m.–12 p.m.), and late night (1 a.m.–4 a.m.). The percentage of kills made by each carnivore in each period was calculated and the exact binomial confidence intervals calculated to compare activity patterns across species.

### 2.5 Prey selection

Patterns of prey selection and the processes that yield them will be analyzed in detail elsewhere, but briefly, we examined dietary overlap using the set of kills just described, plus 101 kills (a total of 554) that could be ascribed to a carnivore but could not be assigned a time of death with sufficient precision for the analysis of temporal activity patterns. The densities of prey species have been estimated (M'Soka et al., 2016), but these data are not directly relevant to a comparison of prey selection by each carnivore species from a single prey community available to them all. Restated, use of each prey species could be converted to use/availability, but the same denominator would be applied to all carnivores, so differences among carnivores would not be affected.

## 3 Results

### 3.1 Prey selection

**Figure 3:**
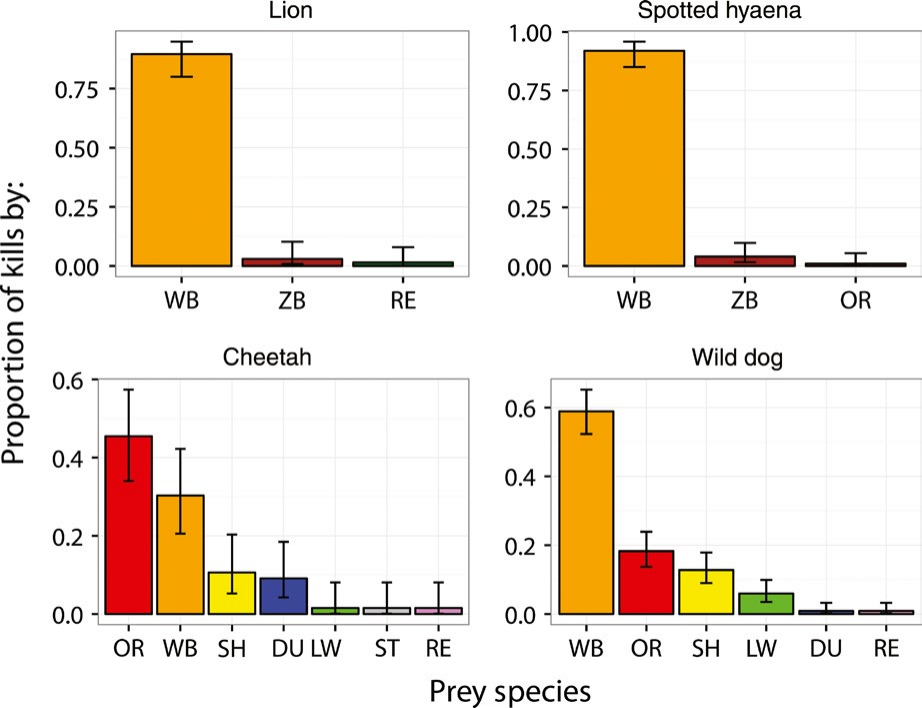
Patterns of prey selection by each of the large carnivore species. WB, wildebeest; ZB, zebra; OR, oribi; SH, springhare; DU, duiker; LW, red lechwe; ST, steenbok; RE, reedbuck

Predation by all four carnivores concentrated on the most common ungulate species, particularly wildebeest (Figure 3). Wildebeest were the most important prey for three of the four carnivores, comprising 92% of hyena kills (95% CI: 85%–96%), 90% of lion kills (95% CI: 85%–96%), 59% of wild dog kills (95% CI: 52%–65%), and 30% of cheetah kills (95% CI: 20%–42%). Lions and hyenas preyed almost exclusively on wildebeest, while cheetahs and wild dogs had broader diets that commonly included oribi, which were very rarely killed by lions or hyenas. Together, wildebeest and oribi comprised 77% of wild dog kills and 74% of cheetah kills.

Thus within LPNP, there is substantial overlap between the diets of all large carnivores, and the potential for interference competition is high, particularly when hunting wildebeest, which are by far the most abundant prey species within the study site.

### 3.2 Spatial niche partitioning

**Figure 4:**
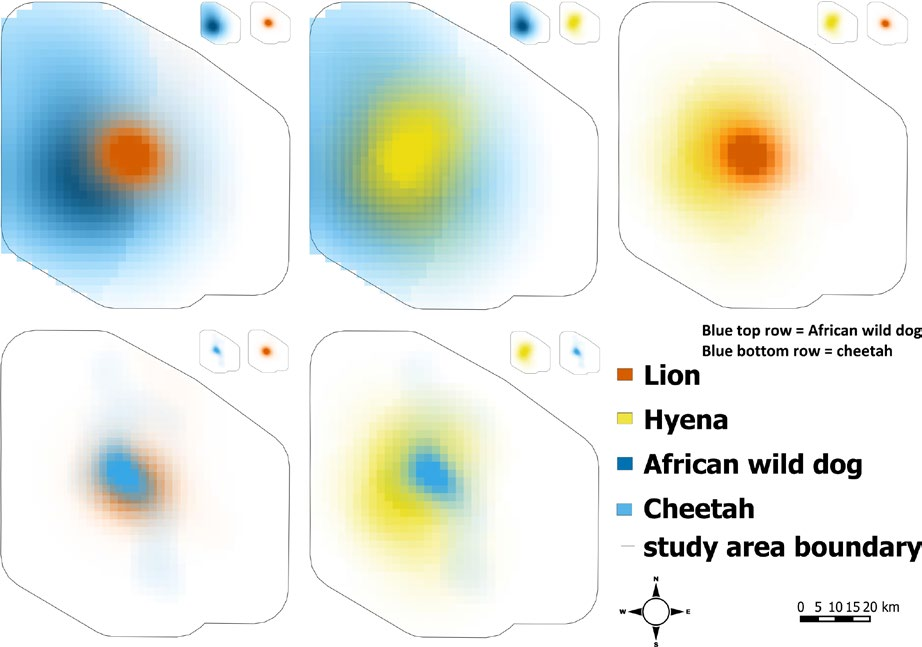
Utilization distributions for each pair of species for which we tested associations. In each panel, the outline denotes the boundaries of the 1,200‐km^2^ focal study area, and the intensity of color indicates intensity of use

Figure 4 shows overlaps between UDs for each pair of species that we examined, within the 1,200‐km^2^ study area. All species concentrated their activities in grasslands in the western portion of the study area (see Figure 2), and rarely used wooded areas to the east.

**Figure 5:**
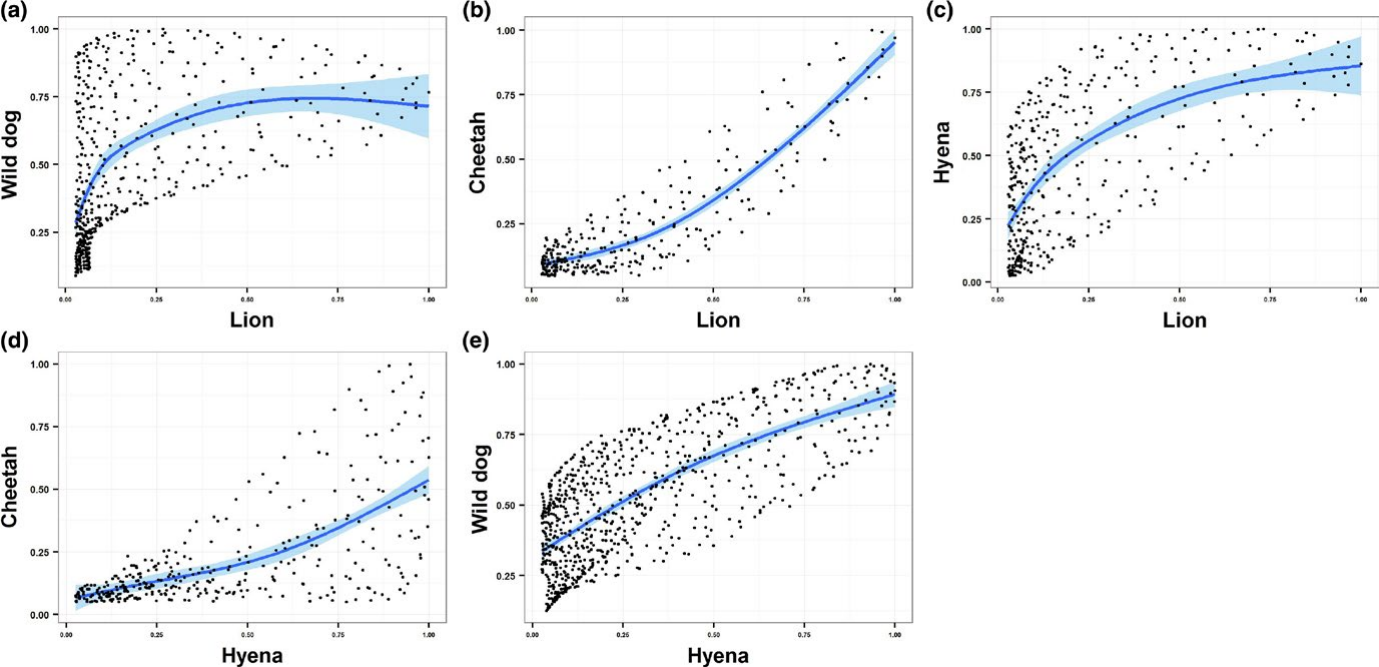
Correlations between space use by species pairs within areas used by both species. (a) wild dog and lion; (b) cheetah and lion; (c) hyena and lion; (d) wild dog and hyena; (e) cheetah and hyena

African wild dog utilization densities were best described by a quadratic response to increasing lion utilization densities (Figure 5a), rather than a linear relationship (drop‐in‐deviance = 8.01, *df* = 1, *p *<* *.0001). The linear component of this relationship was positive (*b *=* *6.45, 95% CI: 5.07–7.85, *t*(484) = 9.125, *p* < .0001), but there was an equally strong negative quadratic effect (*b *=* *−8.14, 95% CI: −6.76 to −3.42, *t*(484) = −5.979, *p *<* *.0001), indicating some degree of avoidance of the areas most heavily used by lions. African wild dog utilization densities increased linearly with increasing hyena utilization densities (Figure 5d, *b* = 2.81, 95% CI: 2.61–3.01, *t*(780) = 27.38, *p *<* *.0001), providing no evidence of spatial avoidance.

**Figure 6:**
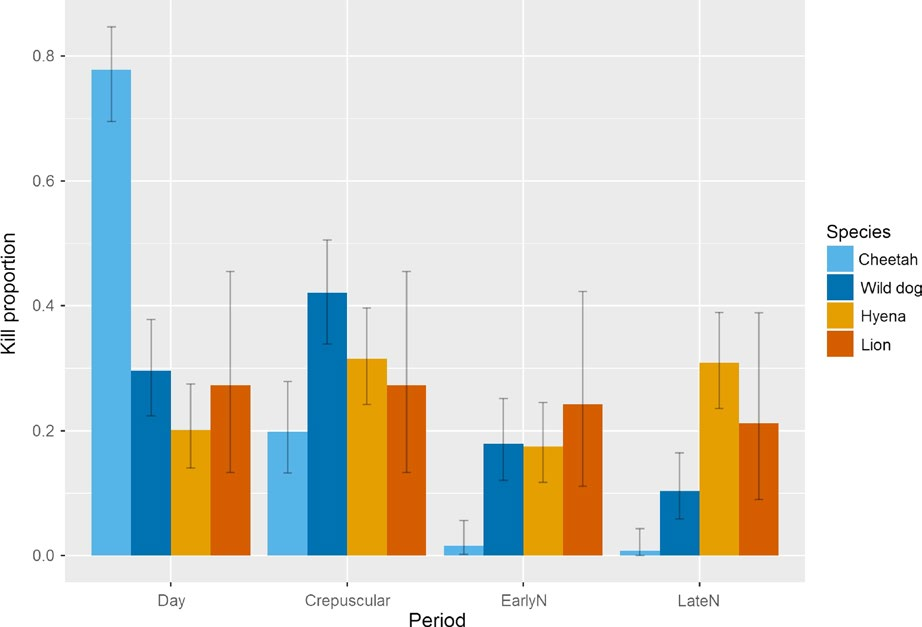
The proportion of kills made by each carnivore species in four time windows. EarlyN and LateN denote early and late night: See section 2 for details. Species color codes are the same as in Figure 4

Cheetah utilization densities increased linearly with increasing hyena utilization densities (Figure 5e, *b* = 2.67, 95% CI: 2.36–2.98, *t*(347) = 17.07, *p* < .0001), and exponentially with increasing lion utilization density (Figure 5b, *b* = 2.61, 95% CI: 2.45–2.77, *t*(282) = 13.575, *p *<* *.0001), providing no evidence of spatial avoidance of either species.

Hyena utilization densities were quadratically related to lion utilization densities (Figure 5c). The linear component of this relationship was positive, as seen above for the the effect of lions on Afrcian wild dogs (*b *=* *6.55, 95% CI: 5.05–8.07, *t*(381) = 8.513, *p* < .0001), with a weaker negative quadratic effect (*b *=* *−3.90, 95% CI: −5.71 to −2.05, *t*(381) = −4.195, *p *<* *.0001) when compared to the responses of wild dogs to lions.

### 3.3 Temporal niche partitioning

Cheetahs made most of their kills during daylight hours (0.70, 95% CI 0.61–0.78), and they had by far the highest proportion of daytime kills of all species. Cheetahs also made a considerable proportion of their kills in the crepuscular periods (0.29, 95% CI 0.21–0.37) with very few kills early in the night (0.01, 95% CI 0.0002–0.04) or late at night (0.01, 95% CI 0.0002–0.040). The largest proportion of kills (0.42, 95% CI 0.34–0.51) by African wild dogs was during the crepuscular periods, and wild dogs made a larger proportion of kills in this period than any of the other species. African wild dogs also made a considerable proportion of their kills during the day (0.30, 95% CI 0.22–0.38) and the early night (0.18, 95% CI 0.12–0.25) but made few kills late at night (0.10, 95% CI 0.059–0.16). The proportion of kills for lions and hyenas were spread more evenly over the time periods. Lions kills were most common in the crepuscular periods (0.36, 95% CI 0.20–0.55) and both night periods (early night 0.24, 95% CI 0.11–0.42, late night 0.24, 95% CI 0.11–0.42), with fewer kills in the daytime (0.15 95% CI 0.05–0.32). Hyenas had their highest proportions of kills during the late night period (0.35, 95% CI 0.27–0.43), and the proportion of kills during the crepuscular, early night, and daytime periods were 0.26 (95% CI 0.19–0.34), 0.23 (95% CI 0.16–0.30), and 0.16 (95% CI 0.11–0.23), respectively.

To summarize, the proportion of kills made by lions and hyenas did not detectably differ across time periods, with appreciable killing rates throughout the entire 24 hr, but with half or more of their kills made in full darkness. Wild dogs and particularly cheetahs each concentrated their hunting into windows that avoided the nighttime hunting window, when both lions and hyenas were still highly active. Cheetahs also had a very low proportion of kills during the early night, when wild dogs, lions, and hyena all showed substantial activity. Thus, the temporal niches of the dominant competitors were broad, while the niches of both the suboridinate competitors (particularly cheetahs) reduced nocturnal overlap.

## 4 Discussion

Niche partitioning is expected to reduce the effects of interspecific competition (particularly for subordinate competitors), and can potentially be accomplished through spatial avoidance, temporal avoidance, or prey selection. We found evidence for all of these mechanisms, with differences among species pairs that yield insights about coexistence and competitive exclusion.

All of the species in LPNP's large carnivore guild show substantial overlap in prey selection with other guild members. Such dietary niche overlap creates the potential for interspecific competition, which for these species can manifest as aggressive interference competition sufficient to cause serious injury or death. As predicted by niche theory, subordinate competitors persist within LPNP in part by widening their dietary niche to avoid the narrower niches of the dominant competitors (Hayward & Kerley, 2008), which specialize almost exclusively on the most abundant resource, wildebeest. Alternatively (or additionally), small prey species such as oribi may provide a higher benefit/cost ratio for the smaller wild dog and cheetah than they do for larger hyenas and lions. Even this mechanism might be related to interspecific competition, because smaller prey species are more likely to be consumed before dominant competitors can detect and aggregate at a kill. The patterns of dietary niche overlap within the carnivore guild of LPNP conform to patterns described across ecosystems, with subordinates showing a “decreasing preference for [common] prey species and increase in number of prey species killed” to reduce niche overlap with dominants (Hayward & Kerley, 2008).

The Liuwa ecosystem has several important ecological properties that affect interactions among large carnivores. First, the vegetation structure is highly uniform within the area we studied, and is typified by open grasslands with good visibility over long distances (Figure 2). Under these conditions, large carnivores can detect one another relatively easily and spatial avoidance is not easily accomplished, particularly when dominant competitors seek out opportunities to steal kills. Second, the ungulate prey community has a simple structure that is highly dominated by wildebeest, with much lower numbers of zebra, oribi, and other species. All of the large carnivores prey heavily on wildebeest, although oribi are also critical prey for wild dogs and especially cheetahs. We suggest that open environments with a simple prey set constrain the options for subordinate species like wild dogs and cheetahs, promoting competitive exclusion. Aligning with this inference, wild dogs have been competitively excluded from ecologically similar areas in Serengeti National Park and the Ngorongoro Crater (Creel & Creel, 1996; Estes & Goddard, 1967; Malcolm & Marten, 1982), and have not been resident within the LPNP study site for more than a year. Our data also suggest that cheetahs may coexist with dominant competitors under these conditions because of more complete temporal niche partitioning to heavily exploit the daylight hours (Swanson et al., 2014).

### 4.1 Relationships of African wild dogs to dominant competitors

Like hyenas and lions, wild dogs relied most heavily on wildebeest in LPNP, but the dominant competitors specialized almost completely on wildebeest, while the dietary niche of wild dogs was much broader, particularly due to predation on oribi. This pattern might be explained by differences in the cost/benefit ratio of hunting smaller prey (Creel & Creel, 2002), but it also aligns with a basic prediction of competition theory, that a subordinate competitor can persist by adopting a generalist strategy that reduces niche overlap with dominant competitors that specialize on the most profitable niche space. Contrary to expectation, space use by African wild dogs was positively related to utilization by lions and hyenas, a result that contrasts sharply with prior comparisons both across ecosystems (Creel & Creel, 1996; Swanson et al., 2014) and within ecosystems (Creel & Creel, 2002; Masenga et al., 2015; Mills & Gorman, 1997; Vanak et al., 2013). In these ecosystems, African wild dogs persisted stably over long periods, while they disappeared from LPNP and other open grassland systems dominated by wildebeest (Estes & Goddard, 1967; Fanshawe & Fitzgibbon, 1993), suggesting that spatial niche partitioning may be critical for competitive coexistence of wild dogs with dominant competitors. Despite the generally positive correlation in space use by wild dogs and dominant competitors, the relationship to relative use by lions was asymptotic while the relationship to relative use by hyenas was linear, even though hyenas outnumbered lions 40‐fold. To some degree, this result aligns with prior studies showing that African wild dogs avoid areas with a high probability of encountering lions.

The difference in spatial relationships of wild dogs to lions and hyenas could perhaps be explained by the fact that a small number of lions in a single pride could be avoided more easily and effectively than the much larger number of hyenas in several clans. The difference might also be explained by recognizing that intraguild predation on wild dogs is more commonly due to lions than to hyenas (Creel & Creel, 1998; Mills & Biggs, 1993) and that wild dogs can dominate hyenas in competitive interactions if they outnumber them (Creel & Creel, 1996; Estes & Goddard, 1967; Fanshawe & Fitzgibbon, 1993). Nonetheless, our results suggest that on a per‐capita basis, the limiting effect of lions on the use of space by wild dogs is strong. We suggest that in an open landscape, it might be impossible for African wild dogs to establish themselves permanently in the face of unavoidable competitors. In other open, relatively intact ecosystems in Africa (Serengeti, Etosha, Ngorongoro), African wild dogs have also been present only intermittently.

Wild dogs showed appreciable overlap with their dominant competitors in temporal patterns of hunting, but were more active during the crepuscular period and less active in full darkness, as in most other systems (Cozzi et al., 2012; Creel & Creel, 2002; Mills & Gorman, 1997; Rasmussen & Macdonald, 2012).

### 4.2 Relationships of cheetahs to dominant competitors

Cheetahs were the only species that did not prey primarily on wildebeest, preferring the much less abundant oribi, and cheetahs had the broadest diets of the four carnivores. As with African wild dogs, these differences can partially be explained by differences in the cost/benefit ratio of hunting small prey, but also align with the hypothesis that cheetahs' dietary niche has evolved to reduce limitation by dominant competitors. Cheetahs focused their activity in areas heavily used by lions in data aggregated over the long term, as has been found elsewhere (Broekhuis et al., 2013; Swanson et al., 2014), and their space use showed a positive linear relationship with that of hyenas. Broekhuis et al. (2013) found that cheetahs avoid lions in space, but in a reactive way (on a short timescale), by positioning themselves further from lions than predicted by a random distribution within areas of shared use (also see Durant, 2000b). For cheetahs, avoidance of both lions and hyenas is typically more clearly related to temporal patterns rather than spatial patterns (Mills & Biggs, 1993). Cheetahs made about 70% of their kills during full daylight, and thereby avoided hunting in the crepuscular and nighttime periods in which the other species made approximately three quarters of their kills, and when surprise encounters at close range are more likely to occur and more likely to escalate.

### 4.3 Relationships between the dominant competitors, hyena and lion

Space use by hyenas was positively correlated to space use by lions, but the relationship was asymptotic, suggesting avoidance of areas most heavily used by lions. Lions and hyenas also showed very similar temporal distributions of hunts. In general, these results align with the inferences from a recent review by Périquet et al. (2014) who noted that these species have both negative and positive influences on each other, so that the net effect is complex and avoidance is weak. Périquet et al. (2014) suggested that finer patterns in selection of prey age–sex classes and processes affecting scavenging opportunities facilitate coexistence between hyenas and lions, allowing both species to concentrate their hunting in areas of high prey availability.

It is increasingly clear that within Africa's large carnivore guild, interspecific competition can be a strongly limiting force for cheetahs and especially African wild dogs. Both species use niche partitioning to reduce the risk of dangerous interactions, but in different ways that appear to have ramifications for coexistence. Wild dogs show more dietary and temporal overlap with dominant competitors. Cheetahs combine divergence in diet, temporal avoidance, and reactive local spatial avoidance to coexist with lions and hyenas in areas of high prey density, even in open habitats. Our results provide new insight into the conditions under which partitioning may not allow for coexistence for one subordinate species, the African wild dog, while it does for cheetah. Because of differences in mechanisms of response, African wild dogs may be more prone to competitive exclusion (local extirpation), particularly in open, uniform ecosystems with simple (often wildebeest dominated) prey communities, where spatial avoidance is difficult.

## Conflict of Interest

None declared.
